# The treatment of chronic depression with cognitive behavioral analysis system of psychotherapy: a systematic review and meta‐analysis of randomized‐controlled clinical trials

**DOI:** 10.1002/brb3.486

**Published:** 2016-05-03

**Authors:** Philip Negt, Eva‐Lotta Brakemeier, Johannes Michalak, Lotta Winter, Stefan Bleich, Kai G. Kahl

**Affiliations:** ^1^ Department of Psychiatry, Social Psychiatry and PsychotherapyHannover Medical SchoolHannoverGermany; ^2^ Department for Clinical Psychology and PsychotherapyBerlin University of PsychologyBerlinGermany; ^3^ Department of Psychology and PsychotherapyWitten/Herdecke UniversityWittenGermany

**Keywords:** Chronic depression, cognitive behavioral analysis system of psychotherapy, meta‐analysis, systematic review

## Abstract

**Background:**

Chronic depression is a severe and disabling condition. Compared to an episodic course, chronic depression has been shown to be less responsive to psychopharmacological and psychological treatments. The cognitive behavioral analysis system of psychotherapy (CBASP) has been developed as a specific psychotherapy for chronic depression. However, conflicting results concerning its efficacy have been reported in randomized‐controlled trials (RCT). Therefore, we aimed at examining the efficacy of CBASP using meta‐analytical methods.

**Methods:**

Randomized‐controlled trials assessing the efficacy of CBASP in chronic depression were identified by searching electronic databases (PsycINFO, PubMed, Scopus, Cochrane Central Register of Controlled Trials) and by manual searches (citation search, contacting experts). Searching period was restricted from the first available entry to October 2015. Identified studies were systematically reviewed. The standardized mean difference Hedges' *g* was calculated from posttreatment and mean change scores. The random‐effects model was used to compute combined overall effect sizes. A risk of publication bias was addressed using fail‐safe *N* calculations and trim‐and‐fill analysis.

**Results:**

Six studies comprising 1.510 patients met our inclusion criteria. The combined overall effect sizes of CBASP versus other treatments or treatment as usual (TAU) pointed to a significant effect of small magnitude (*g *=* *0.34–0.44, *P *<* *0.01). In particular, CBASP revealed moderate‐to‐high effect sizes when compared to TAU and interpersonal psychotherapy (*g *=* *0.64–0.75, *P *<* *0.05), and showed similar effects when compared to antidepressant medication (ADM) (*g *=* *−0.29 to 0.02, *ns*). The combination of CBASP and ADM yielded benefits over antidepressant monotherapy (*g *=* *0.49–0.59, *P *<* *0.05).

**Limitations:**

The small number of included studies, a certain degree of heterogeneity among the study designs and comparison conditions, and insufficient data evaluating long‐term effects of CBASP restrict generalizability yet.

**Conclusions:**

We conclude that there is supporting evidence that CBASP is effective in the treatment of chronic depression.

## Introduction

Depressive disorders constitute a serious public health concern, affecting about 17–22% of the population in its lifetime (Kessler et al. 1997, 2003; Jacobi et al. 2004; Angst et al. 2009). Major depressive disorder is estimated to rank as the 11th greatest contributor worldwide to disability‐adjusted life years (DALY) in 2010, with an increasing impact on DALY's between 1990 and 2010 (Murray et al. 2012). High rates of relapse and recurrence, considerable functional impairments, and protracted lifetime courses frequently occur (Michalak and Lam 2002; Wittchen and Jacobi 2005; Mondimore et al. 2007). The estimated lifetime risk of suicide among patients with major depression is 3.7% for women and 6.7% for men (Nordentoft et al. 2011).

A significant proportion of the individuals suffering from a major depressive episode develop a chronic condition. It is estimated that about 30% of depressed individuals and 47% of the patients presented in mental healthcare services suffer from chronic forms of depression (Arnow and Constantino 2003; Gilmer et al. 2005; Torpey and Klein 2008; Satyanarayana et al. 2009). Furthermore, a number of studies revealed that chronic subtypes of depression are less responsive to traditional treatment approaches (Klein et al. 2008; Schlaepfer et al. 2012).

There is evidence to suggest that episodic and chronic depression might be different in particular characteristics (Murphy and Byrne 2012). Compared to an episodic course, chronic depression is often associated with an early age of onset (<21 years), higher rates of abuse and other adverse experiences (Klein et al. 1999; Klein and Santiago 2003), considerably more comorbid disorders (Keller et al. 1997; Angst et al. 2009), and poorer social adjustment (Ley et al. 2011). Chronically depressed individuals more frequently failed to benefit from psychotherapy and antidepressant medication (ADM) (Keller and Boland 1998; Kocsis 2003), or need to receive higher dosages to improve (Cuijpers et al. 2010a). Due to the growing evidence underlying differences in episodic and chronic courses of depression, in DSM‐5 (American Psychiatric Association, 2013) the diagnosis of persistent depressive disorder has been supplemented. There are beneficial treatments for episodic major depressive disorder available. Of the psychological treatment approaches, cognitive behavioral therapy (CBT), behavioral activation (BA), interpersonal psychotherapy (IPT), and short‐term psychodynamic psychotherapy (STPP) have the strongest empirical support for episodic depression (Butler et al. 2006; Ekers et al. 2008; Driessen et al. 2010; Cuijpers et al. 2011; Barth et al. 2013). However, the evidence base of these treatments in chronic depression is much more limited yet (Berger et al. 2009).

Cognitive behavioral analysis system of psychotherapy (CBASP) has been developed as a specific treatment for chronic major depression (McCullough 2000). CBASP is foremost a behavioral analytic therapy. Behavior and consequences of behavior are the primary targets of CBASP. McCullough posits that chronically depressed individuals have the social functioning characteristics of preoperational children, leading to deficits in social problem solving and interpersonal communication, and links this with the influence of early adverse events (McCullough 2003). Central to CBASP is to teach patients to become connected with the deleterious and depressiogenic consequences of their interpersonal behavior. Specific situational analysis (SA) is the core technique in CBASP, recommended to occupy 80% of treatment time. Other techniques comprise conducting a significant other history (SOH; to teach patients how adverse life experiences still impact their current life), disciplined personal involvement (DPI), and application of interpersonal discrimination exercises (IDE; to encourage patients distinguishing interpersonal reactions of early abusive or neglectful caregivers from reactions of current reference persons). Being originally developed for individual therapy, CBASP has been modified for group formats (Brakemeier et al. 2011, 2015; Michalak et al. 2015).

Initial studies of CBASP provided conflicting results. The combination of CBASP and ADM (nefazodone) significantly increased efficacy and yielded higher remission rates compared to ADM or CBASP as standalone treatments (Keller et al. 2000). These promising results were called into question by another large study, in which CBASP did not outperform brief supportive therapy or antidepressant monotherapy in non‐ or partial responders to pharmacotherapy (REVAMP) (Kocsis et al. 2009). However, these results need to be cautiously interpreted. First, patients in the REVAMP trial had a strong preference of ADM. It is well known that outcome results can be influenced by patients' treatment preference (Kwan et al. 2010; Steidtmann et al. 2012; Gaudiano et al. 2013). Second, individuals assigned to the CBASP condition received on average 12 treatment sessions. A recent meta‐analysis indicated that at least 18 treatment sessions are needed to achieve sufficiently beneficial treatment effects in chronic depression (Cuijpers et al. 2010a). Based on a network meta‐analysis that compared the efficacy and acceptability of several treatment approaches for chronic depression, a recommendation was made of CBASP over IPT (Kriston et al. 2014). Meanwhile, the application of CBASP as a treatment option for chronically depressed individuals has been continued in in‐ and outpatient settings. So far, there is no systematic review and meta‐analysis specifically addressing CBASP. In view of the above, it is of particular interest to investigate the recent efficacy of CBASP in terms of a research synthesis. Our aim is to conduct a systematic review and early meta‐analysis on the current state of evidence of CBASP in chronically depressed patients.

## Methods

The systematic review and meta‐analysis were carried out in accordance with the PRISMA guidelines (Moher et al. 2009).

### Search and study selection

**Figure 1:**
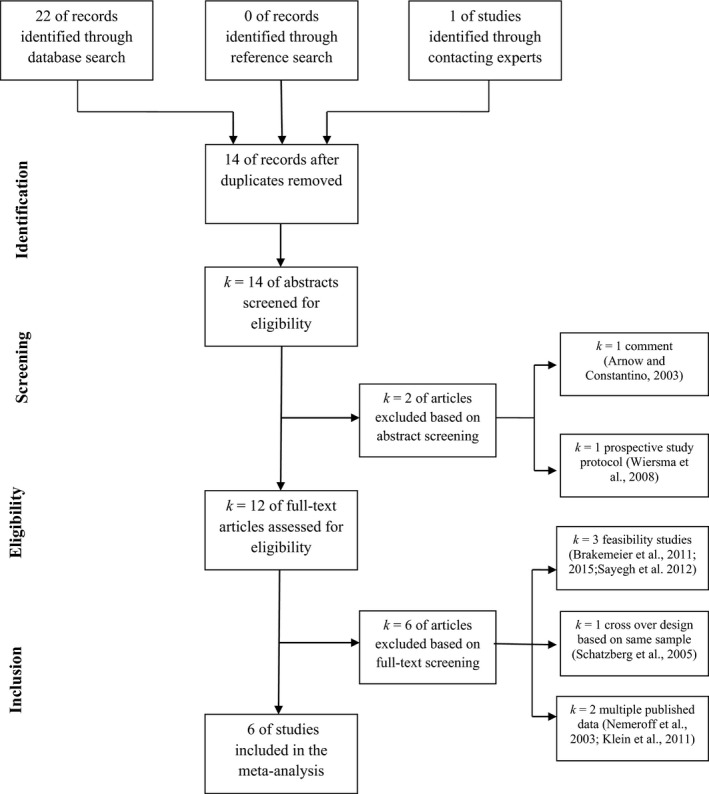
Flow chart of the study selection process following guidelines of Preferred Reporting Items for Systematic reviews and Meta‐analyses (PRISMA; Moher et al. 2009). Reasons for excluding studies from the meta‐analysis are described.

Figure 1 illustrates the process of study selection. A broad literature search was conducted by using the following strategies. First, electronic databases (PsycINFO, PubMed, Scopus, and Cochrane Central Register of Controlled Trials) were browsed. By combining search terms indicative for randomized‐controlled trials investigating the efficacy of CBASP in chronically depressed individuals, eligible studies were retrieved: cognitive behavioral analysis system of psychotherapy*OR*CBASP*AND*depression*OR*major depressive disorder*OR*chronic depression*OR*persistent depression*OR*persistent depressive disorder*OR*dysthymic disorder*OR*dysthymia*AND*randomized*OR*control*OR*trial. Searching period was restricted from the first available entry to October 2015. To give an example of the database search, by browsing PsycINFO 48 records could be identified (retrieved, 30 October 2015). Based on title screening of the results obtained from the databases, a preselection of potentially relevant records was made (22 records). Second, a reference and citation hand search was supplemented. Accordingly, reference lists of identified trials and systematic reviews on third‐wave approaches of CBT or chronic depression were searched for further studies. Third, distinguished experts in the field were asked to verify the completeness of study selection. Thereafter, studies were screened for eligibility on an abstract basis. If the abstract suggested that the study examined clinical outcome results of CBASP – at this stage independent of the specific study design –, the study was read in detail. A coding of central study characteristics was conducted on a full‐text basis. Overall, 12 studies were coded, of which six were excluded from the review (for reasons see Fig. 1, PRISMA flowchart).

### Inclusion and exclusion criteria

To be included in the review, studies had to conform to the following criteria: (1) the treatment under investigation corresponded to the CBASP protocol (McCullough, 2000), (2) the study design was a randomized and controlled clinical trial, (3) standardized diagnostic procedures according to the Diagnostic and Statistical Manual of Mental Disorders (American Psychiatric Association, 2000) were conducted, (4) individuals participating in the study suffered from a chronic course of depression (such as persistent depressive disorder, dysthymic disorder, double depression, or recurrent depressive disorder with incomplete interepisode remission), (5) established outcome measures of depression needed to be used (e.g., Beck Depression Inventory [BDI]; Hamilton Rating Scale of Depression [HAM‐D]) (Hamilton 1967; Beck et al. 1988), and (6) the sample included at least 10 participants in each group. If multiple publications were based on the same research sample, the one reporting the primary depression outcome most comprehensively was selected. Nonrandomized studies, such as quasi‐experimental designs, pre–post comparisons, and case or feasibility studies were excluded from the analysis.

### Data extraction

To extract data from individual studies, a simple coding form was used. The following data were encoded for each study separately: (1) overall sample size, gender distribution, age; (2) comparison conditions, outcome measures used, time point when the outcome was administered, treatment duration, dosage, and format; (3) definitions of methods to ensure therapists' treatment fidelity, clear identification of attrition rates for each treatment and comparison group, explicit descriptions of methods to account for attrition (e.g., ITT, intention‐to‐treat); and (4) means and standard deviations of each available depression outcome and data point (e.g., baseline, posttreatment, follow‐up), remission rates as defined in the individual studies. Data extraction was conducted by the first author (P.N.) and independently verified by the senior investigator (K.K.). In case of any discrepancy concerning the extracted data, a consensual decision was made.

### Multiple outcomes and multiple‐group comparisons

All of the included studies used established outcome measures of depression, either clinical observer ratings, self‐reports, or both. However, if the individual studies under review produce multiple‐effect sizes by using more than one depression outcome (e.g., BDI, HAM‐D), the effects cannot be considered independent of one another (Timm 1999; Morris and DeShon 2002). Therefore, a common method was used in which two outcomes of each individual study (e.g., BDI, HAM‐D) were incorporated in one effect size (Dunlap et al. 1996; Glaser and Olkin 2009). By averaging the effects across all available depression outcome measures, a mean effect size for each study was computed. Following this approach a potential selection bias of favorable effects based on certain outcome measures (e.g., clinical ratings, self‐reports) is being avoided.

The same problem occurs if a single study produces multiple effect sizes by conducting multiple group comparisons (e.g., CBASP vs. control I, CBASP vs. control II). Therefore, effect sizes resulting from different treatment comparisons were averaged to a mean effect size, illustrating the mean effect of CBASP versus other treatment conditions for each study. These synthetic effect sizes were used to aggregate the effect sizes across studies. Only in cases where CBASP was contained in more than one treatment group (e.g., CBASP + ADM vs. CBASP vs. ADM), the effect size comparing the combined treatment to antidepressant monotherapy was considered in the meta‐analysis. Other differences among the treatment conditions were reviewed systematically. By using the above averaging procedures, only one effect size per individual study contributes to a combined effect size.

To investigate whether and how averaging within‐study effect sizes across outcomes and comparison conditions influenced the meta‐analytical results, a sensitivity analysis was conducted. Calculations of combined overall effect sizes were rerun by taking into consideration only effect sizes obtained from (1) the first primary outcome of each study (e.g., HAM‐D), (2) active psychological treatments as comparison conditions (e.g., MBCT, mindfulness‐based cognitive therapy, BSP, brief supportive psychotherapy), and (3) less actively structured comparison conditions (e.g., TAU, treatment as usual, ADM). Accordingly, we were able to examine how these changes might affect the strength of the overall combined effect size.

### Statistical analysis

In order to calculate effect sizes and combine the treatment effects across studies, Comprehensive Meta‐Analysis Version 2.0 (CMA, 2.0, Biostat. Inc., Englewood, Chicago, IL) was used. Studies predominantly used continuous outcome measures of depression, as a result of which the standardized mean difference was determined as the effect size index. As it corrects for a slight upward bias of effect sizes in small samples, Hedges' *g* was chosen. Posttreatment scores of the primary depression outcomes were divided by the pooled standard deviation at posttreat. Additionally, effect sizes based on mean changes from pre‐ to posttreatment were calculated. Mean change scores of the treatment and comparison condition were divided by a pooled standard deviation. The effect sizes illustrated the strength of the treatment effects in terms of symptom severity of depression, with positive effect sizes suggesting advantages of CBASP.

Thereafter, combined overall effect sizes were computed. The duration of treatment considerably varied among the included studies. Due to the fact that all of the studies reported an outcome endpoint, which was administered directly posttreatment, the individual effect sizes based on this endpoint were used to combine the effects across studies. Studies consisting of larger sample sizes produce effect sizes that were more precise estimates of a population effect. Accordingly, each effect size was weighted by the inverse of its variance (Shaddish and Haddock 2009). As only one study reported 1‐year follow‐up data, we were not able to calculate a combined follow‐up effect size. This single effect size was reported in the systematic review section.

Before combining effect sizes, a distinction must be made between fixed‐effect and random‐effects models (Borenstein et al. 2010). Under a fixed‐effect model it is assumed that the effect sizes only differ in terms of sampling errors. In contrast, under a random‐effects model it is assumed that the variability of the observed effect sizes is not only derived from sampling error alone but also from additional sources (i.e., particular study characteristics). With regard to the current sample of studies, there were differences in certain characteristics (i.e., outcome measures, comparison conditions, individual vs. group format). Therefore, the combined overall effect sizes were computed based on random‐effects assumptions. As indicators for heterogeneity among the included studies, the *I* ^2^ and the *Q* statistic were calculated, respectively.

### Risk of bias

The following main characteristics were used to assess a risk of bias in individual studies (Higgins and Green 2008): (1) Methods to ensure treatment fidelity and adherence to the CBASP protocol were adequately described, (2) blinding of outcome assessors was ensured, and (3) attrition rates in each treatment group (CBASP, control) were reported and this information was taken into consideration in the overall analysis (e.g., ITT analysis).

Although efforts were made to identify unpublished studies (i.e., dissertations, conference contributions), the final set of outcome trials entirely consisted of published journal articles. It is known that published and unpublished studies often differ in effect size and statistical significance of the study results – usually referred to as publication bias (Sutton et al. 2000; Onishi and Furukawa 2014). To assess the impact of this bias on the combined overall effect sizes, fail safe *N* procedures (Rosenthal 1979; Becker 2005) as well as trim‐and‐fill analyses (Duval and Tweedie 2000a,b) were conducted.

## Results

### Characteristics of included studies and descriptive analyses

A total of 1.510 subjects were included in the six studies that met our inclusion criteria. Sample sizes of the individual studies varied from 30 to 681. The final set of outcome trials was entirely published in peer‐reviewed journals. Three of the studies were multicenter trials, two of them conducted in the USA (Keller et al. 2000; Kocsis et al. 2009) and one in the Netherlands (Wiersma et al. 2014). Two more were bicentric (Michalak et al. 2015; Schramm et al. 2015). These two studies, and another one, were carried out in Germany (Schramm et al. 2011a). Of the six studies, two investigated the efficacy of CBASP, ADM, and their combination (Keller et al. 2000; Schramm et al. 2015), one compared CBASP to BSP, both as augmentation strategy of pharmacotherapy and antidepressant monotherapy (Kocsis et al. 2009), another one used care‐as‐usual (CAU) psychotherapies applied in the Netherlands (CBT, IPT, short‐term psychodynamic therapy) as comparison condition, both conditions with algorithm‐based pharmacotherapy (Wiersma et al. 2014). One study compared CBASP as monotherapy with IPT as monotherapy (Schramm et al. 2011a). Another study compared a group version of CBASP with MBCT (Segal et al. 2001) and TAU (Michalak et al. 2015). The psychotherapies (CBASP, MBCT) in this trial were augmented to TAU.

There were some variations in the particular study designs, with one of the studies clearly identified as randomized pilot study (Schramm et al. 2011a) and another one that aimed at examining the effectiveness of CBASP in routine practice (effectiveness trial) (Wiersma et al. 2014). All except one study (Schramm et al. 2011a) involved medication.

A majority of the patients were middle‐aged (range = 40.2–50.9; Mdn = 43.3) females (range = 54–65%: Mdn = 58%) with a history of chronic depression (chronic major depression: range = 31–83%; dysthymia or double depression: range = 6–63%; recurrent major depression with incomplete remission: range = 7–23%). Patients received treatment sessions varying from 6 to 25 (Mdn = 19). Participants contained in the REVAMP trial were solely non‐ or partial responders to pharmacotherapy (Kocsis et al. 2009), whereas in another study the whole sample consisted of individuals suffering from early‐onset chronic depression (Schramm et al. 2011a).

Attrition rates among the included trials were 10–28% (Mdn = 22*)*. Five of the six studies statistically analyzed attrition rates and results were presented using the ITT samples. The implementation of methods to ensure adherence to the CBASP protocol and competence of delivery was clearly reported by all of the six studies. Remission rates were 19–57% (Mdn = 35) in the CBASP conditions as compared to 6–50% (Mdn = 25) in the control conditions. Only one study reported 1‐year follow‐up data (Schramm et al. 2011a). Four of the studies used multiple outcome measures (e.g., BDI, HAM‐D), whereas one, respectively, used either the inventory of depressive symptomatology (IDS) (Rush et al. 1996) or HAM‐D. In addition to the posttreatment evaluation, in four studies the outcomes were also administered during treatment phase (Keller et al. 2000; Kocsis et al. 2009; Wiersma et al. 2014; Schramm et al. 2015). A selection of descriptive characteristics of the studies under review is depicted in Table 1.

**Table 1:** Selected descriptive characteristics of the CBASP studies included in the review

Study	Sample size^a^	Women (%)	Age (years)	Adherence (yes/no)	Attrition (%)	Outcome endpoint (week)^b^	ITT analysis (yes/no)	Duration (weeks)	Treatment	
									Dosage (sessions)	Format
Keller et al. (2000)	681	65.3	43	Yes	24.3	12	Yes	12	16	Individual
Kocsis et al. (2009)	491	55.4	44.5	Yes	13.8	12	–	12	12.5	Individual
Schramm et al. (2011a)	30	55	40.2	Yes	10	16	Yes	16	22	Individual
Wiersma et al. (2014)	142	60	41.5	Yes	25	52	Yes	52	25.1	Individual
Schramm et al. (2015)	60	54	43.6	Yes	19	32	Yes	36	22	Individual
Michalak et al. (2015)	106	62.3	50.9	Yes	28.2	8	Yes	10	6–10^c^	Group

ITT, intention‐to‐treat; CBASP, cognitive behavioral analysis system of psychotherapy.

a Overall sample size of participants randomized to treatment and control conditions.

b To aggregate effect sizes across studies, this primary outcome endpoint was used.

c Two initial sessions were conducted individually. –: insufficient information provided.

### Systematic review of the evidence of CBASP

The first randomized‐controlled clinical trial of CBASP, conducted by Keller et al. (2000), revealed that the combination of CBASP and ADM (nefazodon) in chronically depressed participants was more effective in reducing depressive symptoms than both CBASP alone (*g* = 0.54, SE = 0.10, CI_95_ [0.35–0.73], *P *<* *0.001) and antidepressants alone (*g* = 0.49, SE = 0.10, CI_95_ [0.31–0.68], *P *<* *0.001). No differences were found between the two monotherapies (*g* = 0.04, SE = 0.10, CI_95_ [−0.15 – 0.23], *P* = 0.68). The average effect size comparing the combined treatment (CBASP + ADM) to the monotherapies was *g* = 0.52 (SE = 0.10, CI_95_ [0.43–0.82], *P *<* *0.001). The effects calculated from mean change scores produced an effect size of *g* = 0.55 (SE = 0.10, CI_95_ [0.37–0.74], *P *<* *0.001) for the comparison of combined treatment to ADM, and *g* = 0.64 (SE = 0.10, CI_95_ [0.45–0.83], *P *<* *0.001) for the comparison of combined treatment to CBASP monotherapy.

In contrast to the results above, Kocsis et al. (2009) failed to find clear advantages of CBASP augmented to ADM over brief supportive therapy (*g* = 0.18, SE = 0.11, CI_95_ [−0.04 to 0.39], *P* = 0.10) or antidepressant monotherapy (*g* = 0.12, SE = 0.14, CI_95_ [−0.15 to 0.39], *P* = 0.39). Comparing the efficacy of CBASP with the average effect observed in the control groups (brief supportive therapy, ADM), a nonsignificant and small effect size resulted (*g* = 0.15, SE = 0.13, CI_95_ [−0.10 to 0.41], *P* = 0.24). The effect sizes that were calculated from mean change scores of CBASP and BSP (*g* = 0.16, SE = 0.08, CI_95_ [0.01–0.31], *P* = 0.04) as well as CBASP and ADM (*g* = 0.28, SE = 0.10, CI_95_ [0.09–0.48], *P* = 0.004) turned out to be small, but significant, respectively.

A small randomized‐controlled pilot trial was conducted by Schramm et al. (2011a). The comparative efficacy of CBASP and the evidence‐based IPT were investigated and yielded benefits of the CBASP treatment (*g* = 0.75, SE = 0.36, CI_95_ [0.10–1.41], *P* = 0.03). Both treatments were applied as monotherapies without medication. It is important to note that CBASP revealed clear benefits in self‐reported depression (BDI; *g* = 0.85, SE = 0.38, CI_95_ [0.11–1.59], *P* = 0.03), whereas posttreat comparisons of CBASP and IPT in observer‐rated depression were only marginally significant (HAM‐D; *g* = 0.66, SE = 0.37, CI_95_ [−0.07 to 1.39], *P* = 0.08). The effect size derived from the comparison of mean change scores was *g* = 0.62 (SE = 0.34, CI_95_ [−0.05 to 1.28], *P* = 0.07). This was the only study, which provided 1‐year follow‐up data for a considerable number of patients from the original sample (76%). The depression outcomes were administered 12 months after the last therapy session had been conducted. From pretreatment to 1‐year follow‐up, a small sized, but insignificant effect size was observed (*g* = 0.41, SE = 0.43, CI_95_ [−0.44 to 1.25], *P* = 0.35). Approximately one half of this sample received other treatments during the 12 months follow‐up phase (e.g., ADM, CBT, psychodynamic therapy).

In a multisite randomized‐controlled effectiveness trial, Wiersma et al. (2014) compared CBASP to CAU. The control group in this study consisted of evidence‐based treatments (CBT; IPT; short‐term psychodynamic therapy). Both conditions were conducted with algorithm‐based pharmacotherapy. Compared to CAU, chronically depressed individuals assigned to the CBASP condition achieved greater reductions in depressive symptoms at posttreatment (week 52; *g* = 0.55, SE = 0.17, CI_95_ [0.21–0.89], *P* = 0.001). During treatment phase (weeks 8, 16, and 32), CBASP and CAU yielded effects of similar efficacy (*g* = 0.21, SE = 0.17, *P* = 0.22; *g* = 0.21, SE = 0.17, *P* = 0.22; *g* = 0.22, SE = 0.17, *P* = 0.19). Comparing the mean change scores of CBASP and CAU, an effect size of *g* = 0.34 (SE = 0.17, CI_95_ [0.01–0.67], *P* = 0.04) emerged.

A recent trial by Michalak et al. (2015) investigated the comparative efficacy of CBASP and MBCT (Segal et al. 2001), which is another third‐wave approach of CBT specifically addressing the needs of depressed individuals. MBCT was originally developed as relapse prevention for recurrent major depression. Chronically depressed patients either received a group version of CBASP or MBCT, both in addition to TAU, or TAU alone. The results showed clear advantages of CBASP over TAU (*g* = 0.64, SE = 0.22, CI_95_ [0.20–1.08], *P* = 0.004), whereas the effect size obtained from the comparison of CBASP and MBCT proved to be small and insignificant (*g* = 0.18, SE = 0.22, CI_95_ [−2.43 to 0.61], *P* = 0.40). In this sample of chronically depressed patients, MBCT produced an effect size of *g* = 0.42 (SE = 0.05, CI_95_ [0.33–0.52], *P *<* *0.001) compared to TAU. The effect size comparing CBASP to the combined control condition (MBCT, TAU) was of small size and achieved marginal significance (*g* = 0.41, SE = 0.24, CI_95_ [−0.06 to 0.88], *P* = 0.09). The mean change effect size comparing CBASP to TAU was *g* = 0.71 (SE = 0.22, CI_95_ [0.27–1.15], *P* = 0.001). The mean change effect of CBASP and MBCT yielded an effect size of *g* = 0.23 (SE = 0.22, CI_95_ [−0.15 to 0.70], *P* = 0.20).

Finally, Schramm et al. (2015) examined the differential efficacy of CBASP and ADM (escitalopram) as standalone treatments. However, if patients did not improve after 8 weeks of treatment in one of the monotherapy conditions (CBASP; ADM), the other therapy was augmented, respectively. As a result, a more complex data structure arises. From pretreatment to 8‐week posttreatment, CBASP and ADM produced effects of similar efficacy (*g* = −0.29, SE = 0.24, CI_95_ [−0.76 to 0.18], *P* = 0.23). The effect size based on mean change scores was *g* = −0.16 (SE = 0.24, CI_95_ [−0.63 to 0.31], *P* = 0.50). No difference was found between CBASP and ADM. From 8‐week posttreat to 32‐week posttreat, a third treatment group was created which received the combined treatment. Nonimprovers (<20% reduction in depression baseline scores) occurred equally frequent in both treatment conditions. Posttreat effects comparing the combined treatment (CBASP + ADM) to the monotherapies were *g* = −0.41 (ADM; SE = 0.29, CI_95_ [−0.98 to 0.15], *P* = 0.15) and *g* = −0.40 (CBASP; SE = 0.29, CI_95_ [−0.98 to 0.17], *P* = 0.17). Due to the fact that the treatment group receiving the combined treatment had significantly higher depression scores at week 8, these effect sizes must be interpreted with caution. Outcome changes might be higher in the combined treatment group, which is not covered by posttreatment effect sizes in this case. Therefore, effect sizes based on mean change scores are of considerable interest. Comparing the mean changes from 8‐week posttreat to 32‐week posttreat in the combined group (CBASP + ADM) to the mean changes in the ADM group yielded an effect size of *g* = 0.59 (SE = 0.29, CI_95_ [0.01–1.16], *P* = 0.04). Accordingly, the comparison of mean changes in the combined group and the CBASP group produced an effect size of *g* = 0.49 (SE = 0.29, CI_95_ [−0.09 to 1.07], *P* = 0.09). Particularly, the comparison of combined treatment to antidepressant monotherapy produced a medium‐sized mean change effect size, which achieved significance. Central trial characteristics, main results, and mean change effect sizes are depicted in Table 2.

**Table 2:** Trial and outcome characteristics of the six studies investigating the efficacy of CBASP in chronically depressed individuals

Study	Participants	Intervention type (*N*/Attrition‐*N*)	Comparison group (*N*/Attrition‐*N*)	Primary outcome	Remission rates	Main results	Effect size^a^ CI_95_
Keller et al. (2000)	Chronic MDD	CBASP+ (226/47)	ADM [nefazodone] (220/53) CBASP (216/43)	HAM‐D	CBASP + ADM: 48% CBASP: 33% ADM: 29%	Both of the monotherapies (CBASP; ADM) yielded results of similar efficacy, whereas the combined treatment (CBASP + ADM) revealed improved efficacy	*g *=* *0.55 [0.37–0.74] *g *=* *0.64 [0.35–0.73]
Kocsis et al. (2009)	Chronic MDD	CBASP (200/25)	BSP (195/27) ADM (96/16)	HAM‐D QIDS‐CR	CBASP: 43% BSP: 37.9% ADM: 40.6%	None of the therapies (CBASP; BSP) as adjunction to ADM improved the outcome effects over ADM alone. There were no significant differences between CBASP and BSP	*g *=* *0.16 [0.01–0.31] *g *=* *0.28 [0.10–0.48]
Schramm et al. (2011a)	Chronic MDD	CBASP (14/1)	IPT (15/2)	HAM‐D BDI	CBASP: 57.1% IPT: 20%	CBASP and IPT turned out to be equally effective in reducing observer‐rated depression. However, CBASP was significantly superior to IPT in decreasing self‐reported depressive symptoms	*g *=* *0.62 [−0.05 to 1.28]
Wiersma et al. (2014)	Chronic MDD	CBASP (67/16)	CAU (72/19)	IDS‐SR	CBASP: 19.4% CAU: 9.9%	Compared to CAU, patients receiving CBASP revealed greater reductions in depressive symptoms at posttreatment (week 52). No significant differences were found during treatment phase (weeks 8, 16, 32)	*g *=* *0.34 [0.01–0.67]
Schramm et al. (2015)	Chronic MDD	CBASP+ (20/3)	ADM [escitalopram] (16/2) CBASP (17/0)	MADRS IDS‐SR	CBASP: 36.8% ADM: 50% Combined (CBASP + ADM) after nonresponse: 30%	Compared to ADM, individuals assigned to CBASP improved in a similar extent. There were no significant differences. For nonimprovers (after 8 weeks of treatment), the augmentation of the other treatment (ADM, CBASP), respectively, proved to be efficacious	*g *=* *0.59 [0.01–1.16] *g *=* *0.49 [−0.09 to 1.07]
Michalak et al. (2015)	Chronic MDD	CBASP (35/8)	TAU (35/3) MBCT (36/10)	HAM‐D BDI	CBASP: 25.7% MBCT: 16.7% TAU: 5.7%	CBASP was significantly more effective than TAU in reducing depressive symptoms. MBCT failed to reveal clear advantages over TAU across treatment sites. There was no significant difference between CBASP and MBCT	*g *=* *0.71 [0.27–1.15] *g *=* *0.23 [−0.15 to 0.70]

+: specific antidepressant; MDD, major depressive disorder; CBASP, cognitive behavioral analysis system of psychotherapy; MBCT, mindfulness‐based cognitive therapy; IPT, interpersonal psychotherapy; CAU, care‐as‐usual (evidence‐based psychological treatments); BSP, brief supportive psychotherapy; ADM, antidepressant medication; TAU, treatment‐as‐usual; HAM‐D, Hamilton Rating Scale for Depression; BDI, Beck Depression Inventory; IDS‐SR, Inventory of Depressive Symptomatology – Self Rating; QIDS‐CR, Quick Inventory of Depressive Symptomatology – Clinical Rating; MADRS, Montgomery Asperg Depression Rating Scale.

a Effect size (CI_95_): Calculated from mean change scores divided by a pooled standard deviation, comparing CBASP to the control conditions.

### Synthesis of included studies

**Figure 2:**
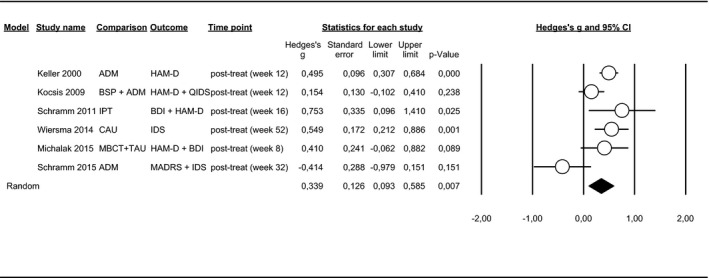
Forest plot of effect sizes (CI95) obtained from six randomized‐controlled trials investigating the efficacy of CBASP in chronically depressed individuals. Posttreatment effect sizes were aggregated under a random‐effects model. CBASP, cognitive behavioral analysis system of psychotherapy; MBCT, mindfulness‐based cognitive therapy; IPT, interpersonal psychotherapy; CAU, care‐as‐usual (evidence‐based psychological treatments); BSP, brief supportive psychotherapy; ADM, antidepressant medication; TAU, treatment‐as‐usual; HAM‐D, Hamilton Rating Scale for Depression; BDI, Beck Depression Inventory; IDS, Inventory of Depressive Symptomatology; QIDS, Quick Inventory of Depressive Symptomatology; MADRS, Montgomery Asperg Depression Rating Scale.

To investigate the more general efficacy of CBASP in chronic depression, a combined effect size was calculated. Posttreatment effect sizes were aggregated using a random‐effects model (Fig. 2). A combined overall effect size of *g* = 0.34 (SE = 0.13, CI_95_ [0.09–0.59], *P* = 0.007) was obtained. Compared to the control conditions, CBASP produced a significant combined effect size of small magnitude. Analysis of heterogeneity among the effect sizes yielded a significant *Q*‐value, which confirmed the use of a random‐effects model (*Q* = 14.27 (5), *P* = 0.014, *I* ^2^ = 64.95).

The overall combined effect size obtained from mean change scores of CBASP and the comparison conditions was *g* = 0.44 (SE = 0.07, CI_95_ [0.31–0.57], *P *<* *0.001; *Q* = 5.11, *I* ^*2*^ = 2.14 *P* = 0.40). According to the analysis of heterogeneity, a nonsignificant *Q*‐value emerged, which might suggest the use of a fixed‐effect model. However, as the *Q*‐test is known to be underpowered in review samples consisting of a small number of studies, a nonsignificant result should not be considered as strong evidence that the effect sizes are consistent among the included trials (Borenstein et al. 2010). Taking into account that the studies used varying control conditions, time points, and outcome measures, the random‐effects meta‐analysis appeared to be more appropriate.

None of the sensitivity analyses considerably altered the overall effect sizes (*g* = 0.36, *P* = 0.002, for first primary depression outcome measures solely; *g* = 0.30, *P* = 0.014 for active psychological comparisons; *g* = 0.37, *P *<* *0.001 for less structured comparisons).

### Assessing the risk of bias

A basic description of methods to ensure therapists' treatment integrity and adherence to the CBASP protocol was provided in each of the six included studies. Accordingly, study therapists continuously received supervision by certified and clinically experienced CBASP trainers. Among all studies, therapy sessions were videotaped or audiotaped and selectively rated for adherence by CBASP supervisors. However, the use of particular adherence rating scales (e.g., CBASP Adherence Scale) was clearly mentioned in only two of the six studies (Kocsis et al. 2009; Michalak et al. 2015). A reporting of the specific results of the adherence ratings indicating nearly excellent treatment integrity was limited to one study (Michalak et al. 2015). Five of six studies clearly reported a blinding of outcome assessors, whereas in one study only a self‐report measure of depression was administered (Wiersma et al. 2014). Moreover, attrition rates and methodological approaches to account for attrition could unambiguously be identified in five of six studies. However, in one study it remained unclear whether attrition rates among the treatment conditions were considered appropriately (Kocsis et al. 2009). With regard to treatment fidelity, blinding of outcome assessors, and handling of attrition, the risk of bias on a study level could be considered as relatively low. Anyhow, the results should be interpreted with some caution as in one study the outcome was restricted to self‐reports of depression and in another study the analysis of attrition remained vague.

Trim‐and‐fill procedures did not indicate influences of publication bias on the combined posttreatment effect size of *g* = 0.34. The effect size adjusted for publication bias turned out to be identical to the combined effect observed in the present meta‐analysis. Classic fail‐safe *N* calculations demonstrated that 32 further studies with nonsignificant or even adverse effects of CBASP would be needed to reduce the combined posttreat effect of *g* = 0.34 to nonsignificance (*P *<* *0.05; *Z* = 1.96). However, the combined mean change effect size of *g* = 0.44 was slightly adjusted due to the risk of publication bias (*g* = 0.43). One adjusted value was imputed through trim‐and‐fill analysis. To bring the combined mean change effect size to nonsignificance, 56 studies with nonsignificant effects of CBASP would need to be added (*P *<* *0.05, *Z* = 1.96.). Nonetheless, as none of the studies included in the current review was retrieved from “grey literature” (e.g., dissertations), it cannot be ruled out that our overall results are slightly affected by publication bias.

## Discussion

The main result of our review is that we found supporting evidence for the effectiveness of CBASP in the treatment of chronic depression by means of a research synthesis. We identified a total of six randomized and controlled clinical trials, in which CBASP was compared to different treatment conditions. In particular, we found a significant combined posttreatment effect size of small magnitude, indicating benefits of CBASP over the control conditions. A significant small‐ to nearly medium‐sized combined effect was obtained when mean change scores of CBASP and the control conditions were compared.

The overall effect sizes observed in our review were smaller than those found in other meta‐analyses on depression treatments: CBT: *d* = 0.67 (Cuijpers et al. 2010b), IPT: *d* = 0.63 (Cuijpers et al. 2011), Short‐term Psychodynamic Psychotherapy: *d* = 0.69 (Driessen et al. 2010). However, it is important to consider characteristics of the specific samples under investigation. All of the individual studies included in the current review comprised subjects suffering from severe chronic and mostly treatment‐resistant forms of depression. In contrast, the above‐mentioned meta‐analyses focused on studies, in which episodically depressed individuals were examined.

Of most interest to our results, a meta‐analysis that explicitly focused on psychological treatments in chronically depressed patients revealed a combined effect size of *d* = 0.23 (Cuijpers et al. 2010a). Accordingly, the authors concluded that psychological treatments yielded small, but considerable effects in chronic depression. In this respect, the significant and increased combined effect size (*g* = 0.34–0.44) among the CBASP studies observed in our review might be considered encouraging.

Compared to the REVAMP trial (12 sessions) (Kocsis et al. 2009), the positive results observed among the other CBASP studies (Keller et al. 2000; Schramm et al. 2011a, 2015; Wiersma et al. 2014) were accompanied by higher dosages of the intervention (16–25 sessions), respectively. Therefore, the dosage or duration of treatment might be a crucial factor in CBASP. In a more recent noncontrolled study, high dosages of CBASP proved to be efficacious in an inpatient setting resulting in high response and remission rates (Brakemeier et al. 2015). However, a considerable number of patients (>60%) exhibited subjective temporary deterioration of symptoms, especially at the beginning of the treatment. It needs to be taken into considerations that in CBASP early adverse childhood experiences are addressed in the initial treatment phase. Later on, the focus of the treatment shifts toward interpersonal problem‐solving skills. The low number of treatment sessions conducted in the REVAMP trial may at least partly explain why CBASP did not show superior effects. Similarly, the results obtained by Wiersma et al. indicated clear advantages of CBASP over other evidence‐based treatments particularly in the longer run (Wiersma et al. 2014). This is in accordance with the results of Cuijpers et al., which demonstrated that approximately 18 treatment sessions are required to achieve favorable results in chronically depressed patients (Cuijpers et al. 2010a). Overall, these results suggest that the individual treatment modality of CBASP may need higher dosages and longer duration to evolve desired effects.

With regard to modifications of the original CBASP approach, the results of Michalak et al. might be promising (Michalak et al. 2015). A group modality of CBASP consisting of two individual and eight group sessions revealed clear advantages over TAU. One factor might be that a group setting facilitates interpersonal learning, which is an important part of CBASP. Despite the fact that CBASP was adapted to the general format of MBCT (8 group sessions of 2.5 h) in this study, it showed benefits. This is worth noting as this low‐dosed group format could be regarded as an unusual treatment approach in chronically depressed individuals. Therefore, it would be of great interest to investigate treatment effects of a higher‐dosage group modality of CBASP. Further studies are required to examine under what circumstances CBASP works most efficiently.

Treatment guidelines for chronic and recurrent major depression recommend a combined modality treatment, which consists of ADM and psychotherapy (Hirschfeld et al. 1997; Bauer et al. 2002; Hegerl et al. 2004). In line with this, two of the included studies showed clearly beneficial results of augmenting CBASP to ADM and vice versa (Keller et al. 2000; Schramm et al. 2015). There is growing evidence to suggest that a combination treatment of psychotherapy and ADM demonstrates advantages over the respective monotherapies in chronic depression (Spijker et al. 2002; De Maat et al. 2007; Huhn et al. 2014; Kriston et al. 2014). Accordingly, a guiding group commissioned by the European Psychiatric Association (EPA) only recently recommended a combination treatment with psychotherapy and pharmacotherapy as the first‐line treatment for chronic depression (Jobst et al. 2016). A meta‐analysis by Karyotaki et al. found superior and enduring effects of a combined treatment modality over ADM monotherapy in major depression. However, according to their results, psychotherapy emerged as adequate alternative to combined treatment during the acute treatment phase of episodic major depression (Karyotaki et al. 2016). Moreover, in a reanalysis of the Keller et al. trial (Keller et al. 2000), evidence has been provided that a crossover from ADM to CBASP was effective for ADM nonresponders, whereas a change from CBASP to ADM resulted in improvements for CBASP nonresponders (Schatzberg et al. 2005). Considering that CBASP is the first psychological treatment approach, which specifically addresses chronic courses of major depression, future research should continue to focus on potentially synergetic effects of CBASP and ADM.

According to the evidence base of CBASP, it is important to consider that all of the studies included in the current review were of relatively high methodological quality, with three of these studies being large multicenter trials (Keller et al. 2000; Kocsis et al. 2009; Wiersma et al. 2014). Comparison conditions at least consisted of TAU. Whereas three of the trials used empirically well‐established active comparisons (CAU, IPT, and MBCT), another two compared CBASP to specific ADM (nefazodone, escitalopram). This is important, as the inclusion of studies that compared active psychotherapies to deleted comparison conditions usually produce higher combined effect sizes. Furthermore, adherence to the CBASP protocol and treatment fidelity was basically monitored among the included trials, which increased explanatory power of the studies. Future studies concerning the efficacy of CBASP should, however, state more precisely whether adherence rating scales were used and how the authors dealt with unsatisfactory treatment integrity.

Following criteria of empirically supported treatments (Chambless and Hollon 1998), the efficacy of a new psychotherapy needs to be supported by at least two randomized‐controlled trials (RCT) of sufficient size and quality, demonstrating superiority to waiting list control conditions, or treatment‐as‐usual, or equal effects to empirically established treatments. Additional evidence should support the method (i.e., single case series or non‐RCTs). Treatments fulfilling these criteria are described as efficacious. Two of the CBASP trials yielded clear benefits over treatment‐as‐usual, or even CAU that comprised mixed evidence‐based treatments (Wiersma et al. 2014; Michalak et al. 2015). Although small in sample size, CBASP outperformed IPT in another trial (Schramm et al. 2011a). There are other study designs supporting the efficacy of CBASP (Brakemeier et al. 2011, 2015; Sayegh et al. 2012; Swan et al. 2014). Moreover, there are currently ongoing multicenter trials of CBASP, in which particular subsamples of chronically depressed patients (early onset) are examined (Schramm et al. 2011b). Accordingly, a further extend of the empirical basis of CBASP is expected. So far, CBASP has not been shown to be inferior to any comparison condition in RCT studies (ADM, TAU, other evidence‐based psychological treatments). Overall, the current state of evidence suggests that CBASP is an efficacious treatment option for chronic depression.

We addressed influences of publication bias on the overall results of our review. It has been recently shown that the assessment of publication bias is underreported in systematic reviews and meta‐analyses (Onishi and Furukawa 2014). This is of particular relevance, as the effects of psychotherapy outcome studies in depressive disorders might be considerably overestimated due to publication bias (Cuijpers et al. 2010b). However, our overall results were only slightly affected by publication bias.

The following limitations of the present review need to be taken into account. Our final set of outcome studies consisted of only six trials. Given that the study pool was heterogeneous in some aspects (e.g., pilot study, effectiveness trial, nonresponders to ADM, sequenced treatments, group vs. individual format, treatment duration), the synthesis of the effect sizes should be interpreted with a certain degree of caution. Moreover, as only one of the included studies reported follow‐up data, the aggregated effect sizes were based on posttreatment outcome assessments. Considering that most chronically depressed individuals suffer from protracted lifetime courses of the disorder, the efficacy of CBASP needs to be proved in the longer run. Consequently, generalizability of the findings is restricted yet. Our results provided initial indications of therapeutic posttreatment efficacy of CBASP by means of a research synthesis.

One might criticize our narrowly defined criteria for including studies. CBASP is a new treatment approach at the stage of efficacy trial testing. Other study designs are essentially important to examine feasibility and acceptability of the CBASP treatment in different settings, formats, and with different populations (Brakemeier et al. 2011, 2015; Sayegh et al. 2012; Swan et al. 2014). However, our aim was to summarize treatment effects in terms of efficacy.

On a more general level the findings of the present review must be considered within the limitations of meta‐analyses. The meta‐analytical results are only as precise as the individual studies on which they are based. For instance, in some of the included studies few details were provided about the comparison conditions (e.g., number of contacts with clinical care providers in TAU conditions, type of ADM, concomitant treatments). As long as important characteristics of the comparison conditions remain vague, it is difficult to draw precise conclusions about the comparative efficacy of a treatment approach.

## Conflict of Interest

The authors declare that there was no conflict of interest concerning this review.
